# Distinguishing hyperfibrinolysis from enhanced–fibrinolytic-type disseminated intravascular coagulation

**DOI:** 10.1016/j.rpth.2024.102574

**Published:** 2024-09-18

**Authors:** Shinya Yamada, Toshihiro Miyamoto, Hidesaku Asakura

**Affiliations:** Department of Hematology, Kanazawa University, Kanazawa City, Ishikawa, Japan

To the Editor,

We read with interest the article by Taylor et al. [1] describing the laboratory tests, treatment, and course of a hyperfibrinolytic condition during the treatment of rhabdomyosarcoma. The authors stated that bleeding during the treatment of rhabdomyosarcoma was due to hyperfibrinolysis, a condition that should be distinguished from disseminated intravascular coagulation (DIC). However, that case was thought to represent DIC with abnormally enhanced fibrinolysis, so-called enhanced–fibrinolytic-type DIC [2].

Differentiating between the 2 requires confirmation using coagulation and fibrinolytic tests of whether coagulation is not enhanced and only fibrinolysis is enhanced or both coagulation and fibrinolysis are enhanced. Generally, thrombin-antithrombin complex (TAT) and prothrombin fragment 1+2 (F1+2) are used as markers of coagulation activation, and plasmin-α_2_ antiplasmin complex (PAP) is used as a marker of fibrinolytic activation.

Conditions in which coagulation is not enhanced and fibrinolysis is solely enhanced include amyloid-light chain amyloidosis [3] and α_2_ plasmin inhibitor (α_2_PI) deficiency [4]. Coagulation tests in patients with these diseases show fibrinogen almost within the normal range and no marked increases in fibrin/fibrinogen degradation products (FDPs) or D-dimer. This is because no consumptive reduction of fibrinogen by excessive coagulation activation occurs, and even if fibrinolysis is enhanced, the amount of lysed thrombus is small. Bleeding in cases with these conditions responds well to antifibrinolytic agents alone [3].

On the other hand, enhanced–fibrinolytic-type DIC shows a marked decrease in fibrinogen and marked increases in FDP and D-dimer [2,5]. Two mechanisms of fibrinogen reduction are involved in enhanced–fibrinolytic-type DIC. The first is consumption due to excessive coagulation activation, while the second is fibrinogenolysis due to excessive fibrinolytic activation. Excessive thrombus formation is dissolved by excessive fibrinolytic activation, resulting in a marked decrease in fibrinogen and marked increases in FDPs and D-dimer. The Figure shows the coagulation and fibrinolytic tests and treatment strategies for hyperfibrinolysis and enhanced–fibrinolytic-type DIC.

**Figure:**
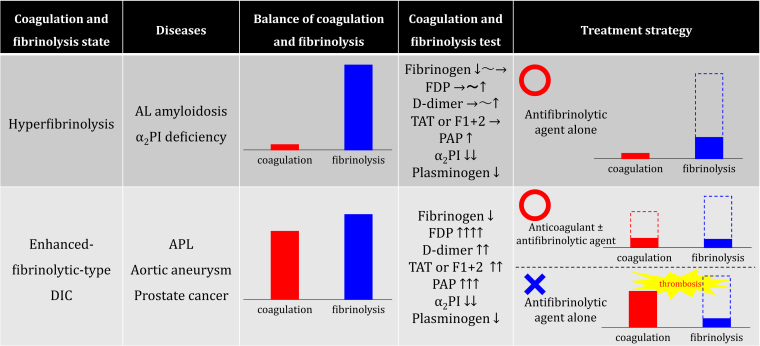
Hyperfibrinolysis versus enhanced-fibrinolytic-type disseminated intravascular coagulation. Typical diseases presenting with each condition, the balance between coagulation and fibrinolysis, coagulation and fibrinolysis tests, and treatment strategies are summarized. Hyperfibrinolysis does not cause excessive coagulation activation and is successfully treated with only an antifibrinolytic agent that suppresses the excessive fibrinolytic activation. Conversely, in enhanced-fibrinolytic-type disseminated intravascular coagulation, excessive coagulation activation and excessive fibrinolytic activation occur simultaneously. Anticoagulation alone or with additional antifibrinolytic therapy should therefore be the treatment strategy. Treatment with an antifibrinolytic agent alone may lead to a predominance of coagulation activation relative to fibrinolytic activation, risking fatal thrombosis. AL, amyloid-light chain; α_2_PI, α_2_ plasmin inhibitor; APL, acute promyelocytic leukemia; FDP, fibrin/fibrinogen degradation product; TAT, thrombin-antithrombin complex; F1+2, prothrombin fragment 1+2; PAP, plasmin-α_2_ antiplasmin complex.

In the article by Taylor et al. [1], in addition to a marked decrease in fibrinogen, both FDP and D-dimer were markedly increased, strongly suggesting enhanced–fibrinolytic-type DIC. To evaluate pathologies of coagulation and fibrinolysis and identify the most appropriate therapeutic intervention, levels of TAT, F1+2, PAP, α_2_PI, and plasminogen should be measured in addition to the common coagulation tests of prothrombin time, activated partial thromboplastin time, fibrinogen, FDP, and D-dimer [5,6]. Decreased activity of factor (F) XIII has also been reported in DIC [7,8].

No standard of treatment has been established for enhanced–fibrinolytic-type DIC, but anticoagulants with or without additional antifibrinolytic therapy are usually used [2,5,6]. Treatment of DIC with antifibrinolytic agents alone is contraindicated because fatal thrombosis is likely to result [9,10]. Indeed, the presence of bleeding may lead to hesitation in the use of anticoagulants. Dose-reduced anticoagulants plus antifibrinolytic therapy is one option for treatment. Fresh frozen plasma instead of cryoprecipitate is another, in terms of balancing the replacement of coagulation and anticoagulation factors. The use of FXIII preparations may be a valid treatment strategy in terms of temporarily staving off local bleeding [6].

As Taylor et al. [1] pointed out, distinguishing between hyperfibrinolysis (excessive activation of fibrinolysis alone) and enhanced–fibrinolytic-type DIC (excessive activation of both coagulation and fibrinolysis) is important to determine the treatment strategy. For this purpose, measuring TAT, PAP, α_2_PI, and plasminogen is necessary. If the authors have preserved samples of residual plasma from this case, we would greatly appreciate measurement of these markers.
